# Enhanced lithium storage performance of porous exfoliated carbon fibers *via* anchored nickel nanoparticles†

**DOI:** 10.1039/c8ra02529k

**Published:** 2018-05-09

**Authors:** Xue Huang, Guiqiang Diao, Siqi Li, Muhammad-Sadeeq Balogun, Nan Li, Yongchao Huang, Zhao-Qing Liu, Yexiang Tong

**Affiliations:** College of Chemistry and Chemical Engineering, Zhongkai University of Agriculture and Engineering Guangzhou 510225 China; School of Chemistry and Chemical Engineering/Guangzhou Key Laboratory for Environmentally Functional Materials and Technology, Research Institute of Environmental Studies at Greater Bay, Guangzhou University, Guangzhou Higher Education Mega Center Outer Ring Road No. 230 Guangzhou 510006 China lzqgzu@gzhu.edu.cn; School of Chemistry and Materials Engineering, Huizhou University 516007 Huizhou PR China; KLGHEI of Environment and Energy Chemistry, The Key Lab of Low-Carbon Chemistry & Energy Conservation of Guangdong Province, MOE of the Key Laboratory of Bioinorganic and Synthetic Chemistry, School of Chemistry, Sun Yat-sen University 135 Xingang West Road Guangzhou 510275 China balogun@mail2.sysu.edu.cn chedhx@mail.sysu.edu.cn; Middle School Attached to Guangzhou University Guangzhou P. R. China

## Abstract

Herein, flexible carbon fiber cloth (CFC) is modified by embedding Ni nanoparticles *via* a thermal reduction strategy, and it is used as a suitable anode material for lithium-ion batteries. Benefitting from the elemental interaction between Ni and carbon, the Ni-embedded CFC displayed higher lithium storage properties than pristine CFC and Ni-free porous CFC.

## Abstract 2

Herein, flexible carbon fiber cloth (CFC) is modified by embedding Ni nanoparticles *via* a thermal reduction strategy, and it is used as a suitable anode material for lithium-ion batteries.

## Graphical abstract

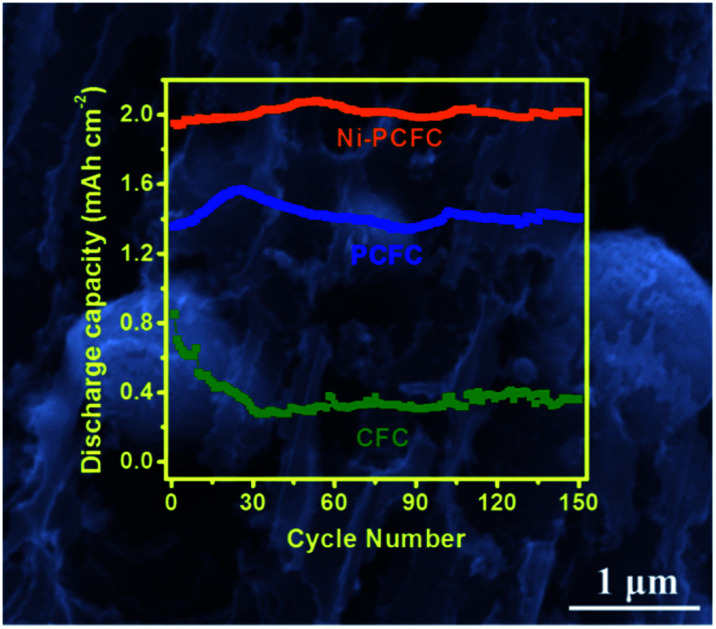

The rapid development of flexible energy storage devices, especially flexible lithium-ion batteries (LIBs), requires urgent development of flexible electrodes.^1–7^ The most common flexible electrodes require the growth of active anode materials, such as carbon cloth, nickel foam, titanium mesh, *etc.*, on flexible current collectors.^8–12^ This strategy is very effective in achieving high capacity flexible anode but still exhibits relatively low volumetric capacity due to the thickness of current collectors.^13^ Another interesting strategy used to develop flexible anode materials is the utilization of different physical and chemical methods to synthesize flexible graphene and carbon nanotubes, which can be used directly as anode materials for LIBs.^14–18^ With substantial improvement and lightweight properties, such flexible electrodes with poor mechanical strength, low flexibility, poor cyclic stability and low rate capability greatly limit the practical applications.^13^ Hence, it is imperative to develop flexible anode materials with good mechanical strength, high flexibility and enhanced lithium storage properties.

Recently, carbon fiber cloths (CFCs) have been employed not only as substrates or current collectors for active materials but also as electrode materials in LIBs,^13^ supercapacitors,^19,20^ and water splitting.^21,22^ Our previous reports have shown that the surface area of CFC can be enhanced *via* thermal etching.^13,21^ However, the capacity obtained is not satisfactory enough. Moreover, the interaction of transition metals with carbon is said to improve the kinetics, introduce more active surface areas on carbon and enhance the power density of energy storage devices.^11,22,23^

Motivated by our previous study, herein, Ni NPs were incorporated in CFC (Ni–PCFC) *via* a thermal reduction strategy, and Ni–PCFC was used as an anode material for LIBs. After thermal reduction and embedment of Ni NPs into CFC, the surface of the sample became porous, which was beneficial for improving its storage performance. Hence, Ni–PCFC exhibited superior lithium storage properties when compared with pristine CFC and Ni–free porous CFC (PCFC). This study can create an opportunity for the use of carbon fiber not only as a flexible anode material for LIBs but also as a capacity contributor when it is used as a current collector for other active electrodes.

Ni–PCFC was synthesized by a simple hydrothermal reaction and annealing treatment. First, Ni(OH)_2_·H_2_O was grown on CFC (eqn (1)). CFC (ESI, Fig. S1† and 1-I) was covered with Ni(OH)_2_·H_2_O nanosheets (Fig. S2† and 1-II). The Ni(OH)_2_·H_2_O–CFC nanosheets were then annealed in N_2_ gas. At an annealing temperature of 500 °C, NiO and Ni were formed (NiO@Ni–CFC), as shown in Fig. 1-III and eqn (2). The scanning electron microscopy (SEM) images of NiO@Ni–CFC are displayed in Fig. S3.† At this point, the nanosheets could still be maintained, but they became porous. By increasing the temperature, the morphology of nanosheets was damaged through thermal reduction, which allowed the carbon of the carbon fiber to pyrolyze, and this also created nucleation and further anchoring of porous sites for the embedment of Ni NPs (Fig. 2a, the enlarged image can be found in Fig. S4†). Ni–PCFC was formed (Fig. 1-IV) according to eqn (3).

1 Ni^2+^ + 2OH^−^ → Ni(OH)_2_

2 Ni(OH)_2_·H_2_O + *x*C + N_2(g)_ → NiO + Ni + C + H_2_O↑

3 NiO + Ni + C + N_2(g)_ → Ni + C + *x*CO↑

**Fig. 1:**
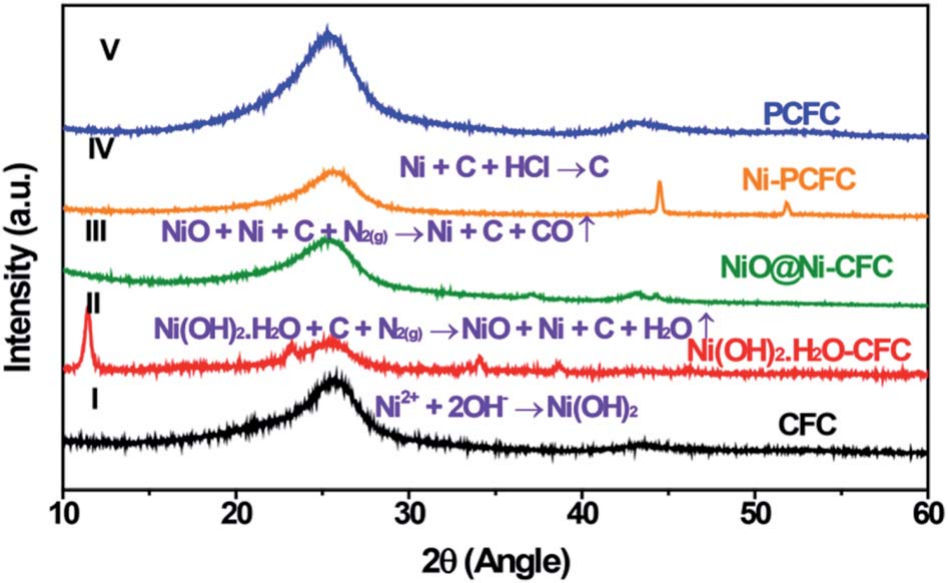
XRD patterns showing the formation of Ni–PCFC and other samples. (I) CFC, (II) Ni(OH)_2_·H_2_O–CFC, (III) NiO@Ni–CFC, (IV) Ni–PCFC and (V) PCFC.

**Fig. 2:**
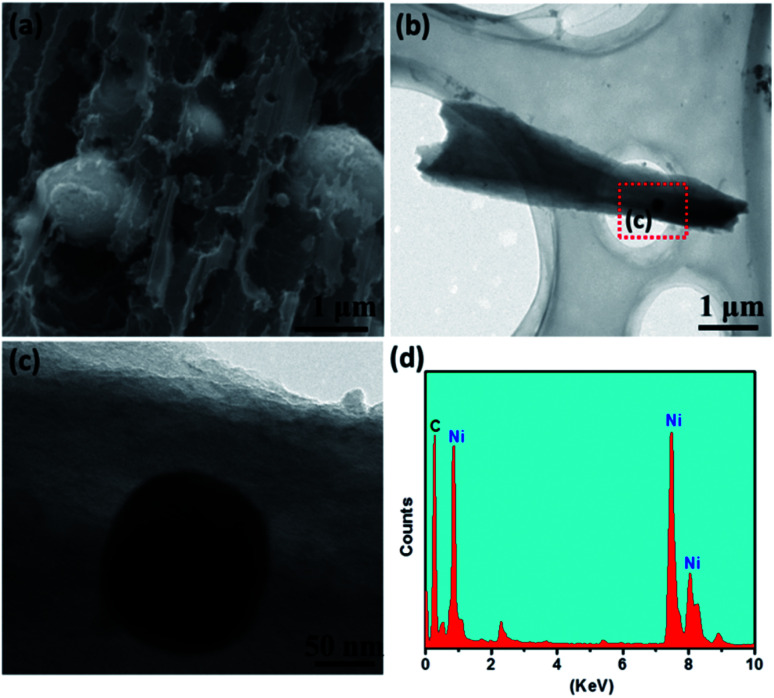
(a) SEM, (b) TEM and (c) HRTEM images of Ni–PCFC. (d) EDS spectrum of Ni–PCFC.

In addition, Ni–PCFC could be etched with concentrated HCl overnight to remove Ni NPs.

4 Ni + C + HCl → C

Hence, the Ni–free porous carbon fiber (PCFC) was formed (Fig. 1-V, 2b and S5†). Transmission electron microscopy (TEM) analysis confirmed that Ni NPs were embedded in CFC (Fig. 2b). High-resolution TEM (HRTEM) image of Ni–PCFC displayed that CFC was porous and exfoliated (Fig. 2c). The energy spectrum (EDS) from Fig. 2c also revealed the presence of both Ni and C, confirming the successful formation of Ni–PCFC. The Ni and carbon contents of Ni–PCFC composite were determined using an inductively coupled plasma mass spectrometer (ICP-MS) and elemental analyzer, respectively. The results showed that the content of Ni was 6.0% in Ni–PCFC, whereas that of carbon was 93.86% in 10 mg cm^−2^ Ni–PCFC sample. Additionally, XPS analysis also showed that the atomic percentage of carbon was 89.78% and that of Ni was 10.22%.

It has been previously reported that the Brunauer–Emmett–Teller (BET) surface area measurement of pristine CFC is within 5–10 m^2^ g^−1^.^19,20^

Compared to the BET surface area of pristine CFC, the BET surface area of Ni–PCFC reaches 109 m^2^ g^−1^ (Fig. 3a) with a pore volume of 0.21 cm^3^ g^−1^ (Fig. S6†), and the values are even higher than the results of our previously reported study (97 m^2^ g^−1^ and 0.13 cm^3^ g^−1^) (Fig. 3a).^13^ This result further suggests that Ni–PCFC can exhibit better electrochemical properties than CFC. According to Raman spectra analysis, the D : G peak of Ni–PCFC (2.34) is also higher than that of CFC (1.59). This indicates that the surface modification and deficiency in Ni–PCFC is a result of Ni, and this can be beneficial for improving storage performance.^13,24^ Additionally, X-ray photoelectron spectroscopy (XPS) analysis confirms the presence of Ni in Ni–PCFC and absence of Ni in CFC (Fig. S7† and 3c). After exfoliation of CFC, the intensity of C–C peak at 284.8 eV in the C 1s XPS spectra of Ni–PCFC is higher than that of CFC. Moreover, Ni–PCFC is characterized with additional HO–C=O peak at 291.0 eV,^19,20,25^ further confirming that Ni–PCFC with suitable features can improve the electrochemical properties.

**Fig. 3:**
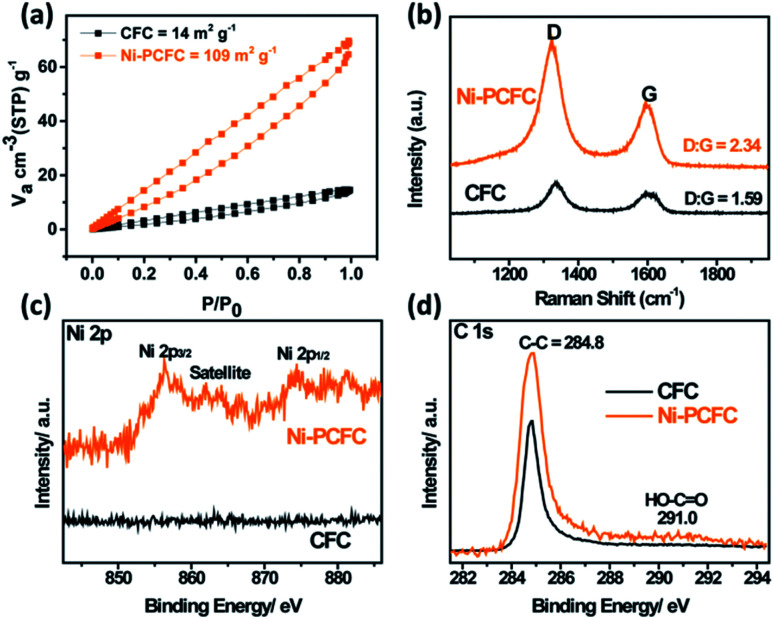
(a) BET surface areas of CFC and Ni–PCFC. (b) Raman spectra, (c) Ni 2p XPS spectra and (d) C 1s XPS spectra of CFC and Ni–PCFC.

The lithium storage properties of Ni–PCFC were tested in a coin cell and compared with those of pristine CFC. The calculated charge/discharge capacities were based on the areas of the electrodes. The charge/discharge capacities were calculated in current × hour per area (*i.e.*, mA h cm^−2^). Ni–free porous CFC (denoted as PCFC) was also tested for comparison to show the contribution of Ni NPs. Detailed results can be found in Fig. 4. The 1^st^ cyclic voltammetry (CV) curve of the electrodes showed that the Ni–PCFC curve area below 1.25 V was much larger than those of PCFC and CFC (Fig. S8a†), indicating the higher electrochemical surface area of Ni–PCFC than those of other electrodes. Additionally, Ni–PCFC was characterized with a cathodic peak at 1.06 V. Such a peak is assigned to the lithiation of Ni with porous carbon^23^ and can lead to additional capacity. During the 2^nd^ cycle (Fig. 4a) and 3^rd^ cycle (Fig. S8b†), larger curve areas and an additional cathodic peak (shifted to 1.31 V) could be observed for the Ni–PCFC electrode.

**Fig. 4:**
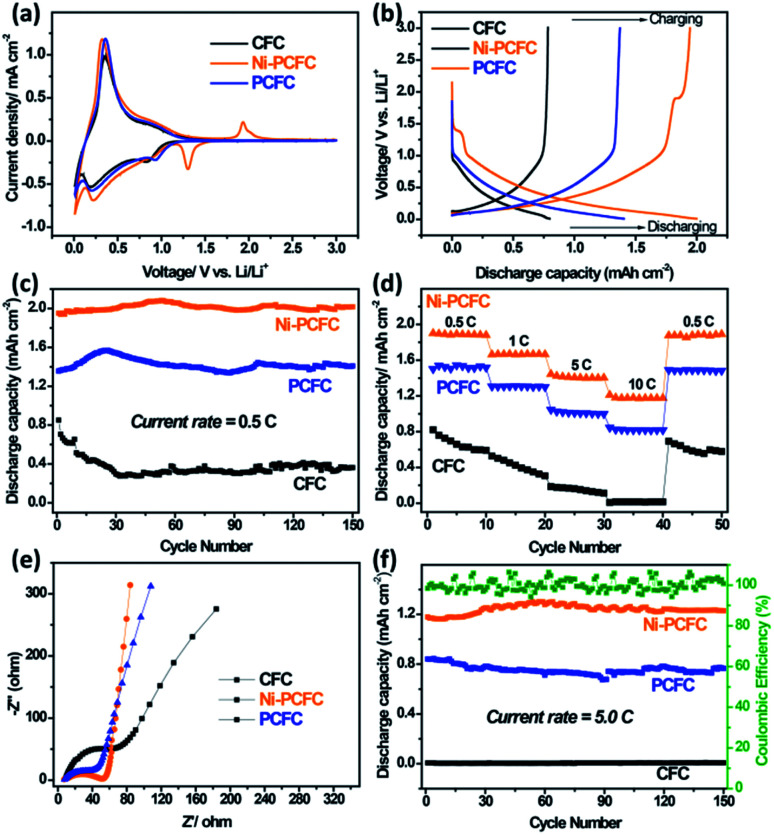
(a) 3^rd^ CV profile and (b) 3^rd^ charge/discharge profile of CFC, Ni–PCFC and PCFC electrodes. (c) Cyclic stability at 0.5C, (d) Nyquist plots, (e) rate capability and (f) cyclic stability at 5C of the CFC, Ni–PCFC and PCFC anodes.

These results indicated the superior storage properties of Ni–PCFC when compared with those of the other electrodes. Such superior capacities could be observed in the charge/discharge profiles of the electrodes. The Ni–PCFC electrode achieved a 2^nd^ cycle discharge capacity of 1.98 mA h cm^−2^, which was higher than those of PCFC (1.48 mA h cm^−2^) and CFC (0.74 mA h cm^−2^) electrodes at a current rate of 0.5C (Fig. 4b). The discharge capacity of Ni–PCFC remained at 1.93 mA h cm^−2^ (Fig. 4c) with 99% coulombic efficiency after 100 electrochemical cycles (Fig. S9†), this value was also higher than those of PCFC (1.34 mA h cm^−2^) and CFC (0.36 mA h cm^−2^). Moreover, the rate capability performance of Ni–PCFC up to 10C was also better than those of PCFC, CFC, (Fig. 4d) and previously reported porous CC.^13^ The outstanding performance can be attributed to the electronic interactions between Ni NPs and exfoliated CF, leading to rapid diffusion of Li ions and electrons, which enhanced the electrode kinetics. This can be confirmed by the smaller charge transfer resistance (*R*_ct_) in the Nyquist plot of Ni–PCFC when compared with those of CFC and PCFC (Fig. 4e). Due to the excellent kinetics of the Ni–PCFC electrode, a higher cyclic performance up to 150 cycles at a current of 5C was obtained, and the Ni–PCFC electrode could deliver a discharge capacity of 1.1 mA h cm^−2^, which was 55% higher than that of PCFC (0.71 mA h cm^−2^) and 99% higher than that of CFC (0.01 mA h cm^−2^). Notably, the XRD spectra of Ni–PCFC before and after electrochemical reactions remained the same, showing excellent phase stability (Fig. S10a†). Moreover, Ni NPs were well anchored on the porous exfoliated surface of CF, further indicating excellent morphological stability of Ni–PCFC (Fig. S10b–d†). Ni and PCFC both contributed towards lithium capacity. To confirm this phenomenon, Ni was dissolved to obtain bare PCFC. The lithium storage performance of PCFC was compared with those of Ni–PCFC and CFC. In Fig. 4a, Ni–PCFC is characterized with a cathodic peak as a result of the anchored Ni NPs, which was totally absent in pristine CFC as well as PCFC. Additionally, according to Fig. 4b–d of our manuscript, it could be observed that Ni–PCFC displayed superior storage performance owing to the anchored nickel. The anchoring of Ni on PCFC created strong electronic interactions between Ni NPs and PCFC, leading to rapid diffusion of Li ions and electrons, which enhanced the electrode kinetics. This indicated that both Ni and PCFC exhibited synergistic effect for enhancing the lithium storage capacity of the Ni–PCFC electrode. Ni–PCFC can be directly used as a flexible anode material for the fabrication of flexible LIBs.

In conclusion, lithium storage properties of carbon fiber cloth (CFC) can be improved by embedding Ni NPs in porous exfoliated CFC (Ni–PCFC) *via* high temperature thermal reduction. Ni–PCFC displays better electrochemical properties than pristine CFC and Ni–free porous CFC (PCFC), due to electronic interactions between Ni NPs and PCFC, additional capacities and higher surface area. This study can create an opportunity for the development of high-capacity carbon-based electrode materials for flexible LIBs.

## Conflicts of interest

There are no conflicts to declare.

## Supplementary Material

RA-008-C8RA02529K-s001
