# Supplementary material for: Enhanced lithium storage performance of porous exfoliated carbon fibers via anchored nickel nanoparticles
Source: RSC Adv. 2018 May 9;8(31):17056–9. doi: 10.1039/c8ra02529k (PMC9080291; doi:10.1039/c8ra02529k)
Supplement: RA-008-C8RA02529K-s001 [file RA-008-C8RA02529K-s001.pdf]

Electronic Supplementary Material (ESI) for RSC Advances.
This journal is © The Royal Society of Chemistry 2018

Supplementary Information

Enhanced Lithium Storage Performance of Porous Exfoliated Carbon Fibers via Anchored Nickel Nanoparticles

Xue Huang,[a] Guiqiang Diao,[c] Siqi Li,[d,e] Muhammad-Sadeeq Balogun,*[d] Nan Li,[b] Yongchao Huang,[b] Zhao-Qing Liu,*[b] and Yexiang Tong*[d]

Experimental Section

Synthesis of the Ni-PCFC.

CFC was purchased from Fuel Cell Earth. All reagents were of analytical grade and were directly used without any purification. Firstly, $Ni(OH)_2.H_2O$ nanosheets (denoted as $Ni(OH)_2.H_2O$-CFC) were grown on the CFC as reported in our previous papers. {Balogun, 2016 #1382} $Ni(OH)_2.H_2O$-CFC was then annealed in N_2 atmosphere at 900 ^oC for 2 h. The furnace was then allowed to cool to room temperature and Ni-PCFC sample was obtained. The Ni-PCFC sample was immersed in conc. HCl overnight to dissolve the Ni nanoparticles and porous PCFC was obtained.

Characterization

The morphology, structure, and composition of the electrode materials were characterized by field emission SEM (JSM-6330F) and transmission electron microscope (TEM) (JEM2010-HR, 200 KV). The element identification and heteroatom functional group distribution was measured through X-ray photoelectron spectroscopy (XPS, ESCALab250). The crystallographic information and phase purity of the products were characterized by X-ray diffraction Spectrometry (XRD; Shimadzu X-ray diffractometer 6000, Cu Kαradiation, Shimadzu, Tokyo, Japan) and Raman Spectroscopy (Renishaw inVia). Specific surface areas of the samples were calculated from a multipoint Braunauer-Emmett Teller (BET) analysis of the nitrogen adsorption and desorption

isotherms at 77K recorded on an Autosorb-1 apparatus. The content of Ni in Ni-PCFC was determined using inductively coupled plasma mass spectrometry-ICP-MS (Optima 8300, PerkinElmer, USA). The content of C in Ni-PCFC was determined by Elemental Analyzer (Elementar Analysensysteme, GmbH).

Electrochemical measurements

The binder free electrodes (CFC, Ni-PCFC and PCFC) were directly used as anode or working electrode. Pristine carbon cloth (CFC) and Ni-PCFC electrodes were cut into many smaller square pieces with the diameter of area of 1.0 cm^2. The charge/discharge capacities are calculated in current*hour per area (i.e. mAh cm^{-2}). Half cells were assembled in a coin cell using 1 M $LiPF_6$ in ethylene carbonate (EC) and diethyl carbonate (DEC) [(1:1)] solution was used as the electrolyte, Celgard 2400 was used as the separator and Li foil as both counter and reference electrodes. The galvanostatic charge/discharge tests were carried out between 0.01−3.0 V vs. Li/Li^+ (anode at different C rates, 1 C=372 mAh/g), on a Neware Battery Testing System (Shenzhen, China).

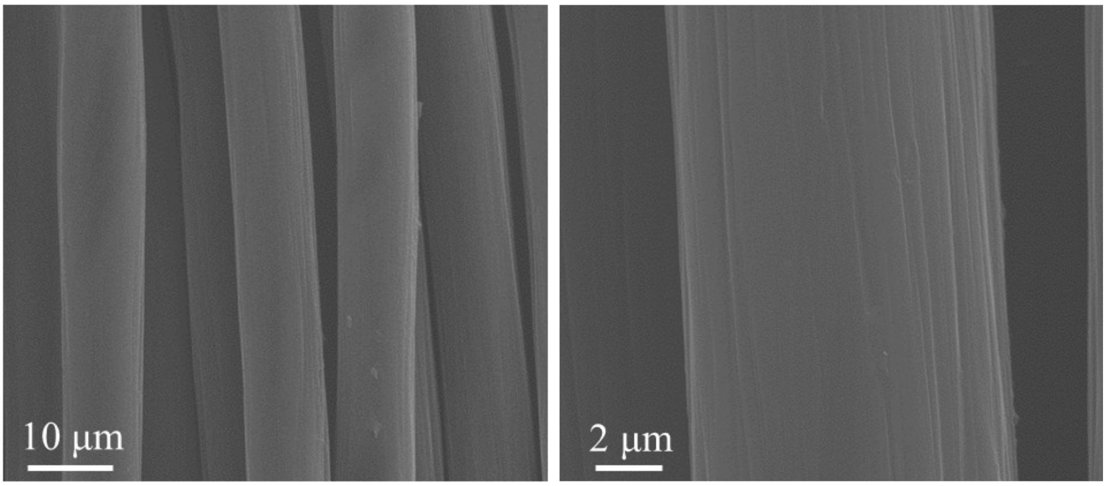

Figure S1. SEM images of CFC.

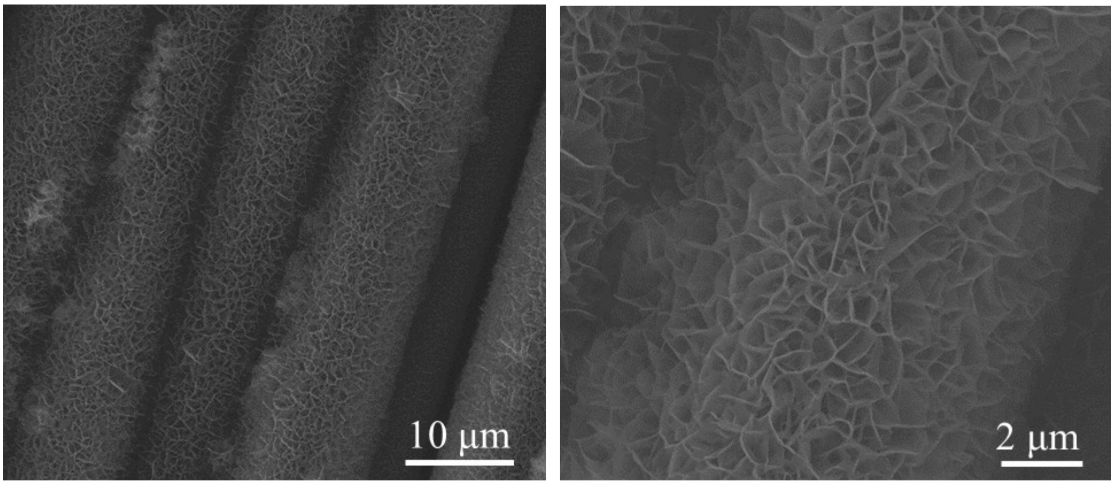

Figure S2. SEM images of $Ni(OH)_2.H_2O$-CFC.

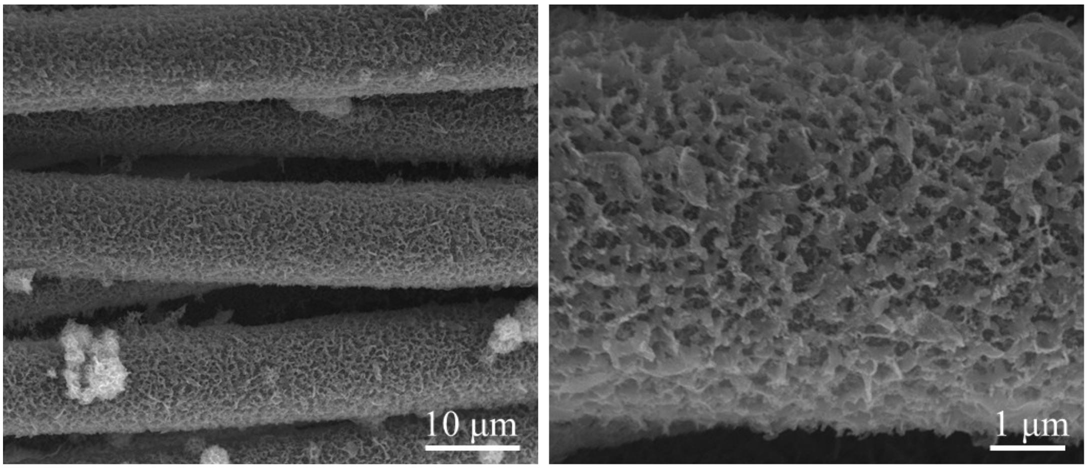

Figure S3. SEM images of NiO@Ni-CFC.

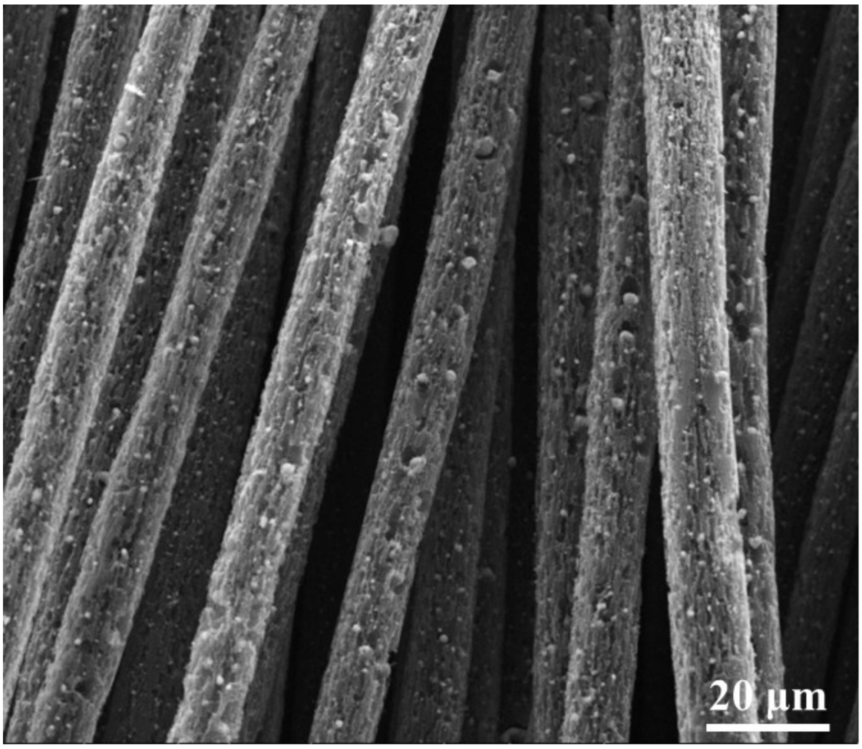

Figure S4. Enlarge SEM image of Ni-PCFC.

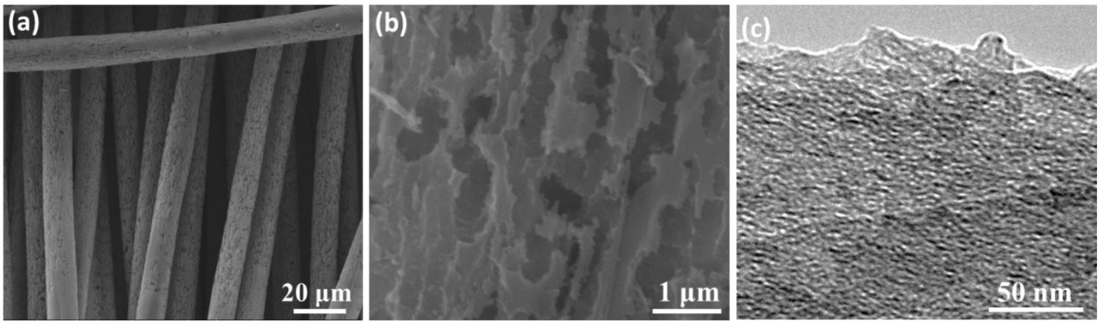

Figure S4. Morphological images of PCFC.

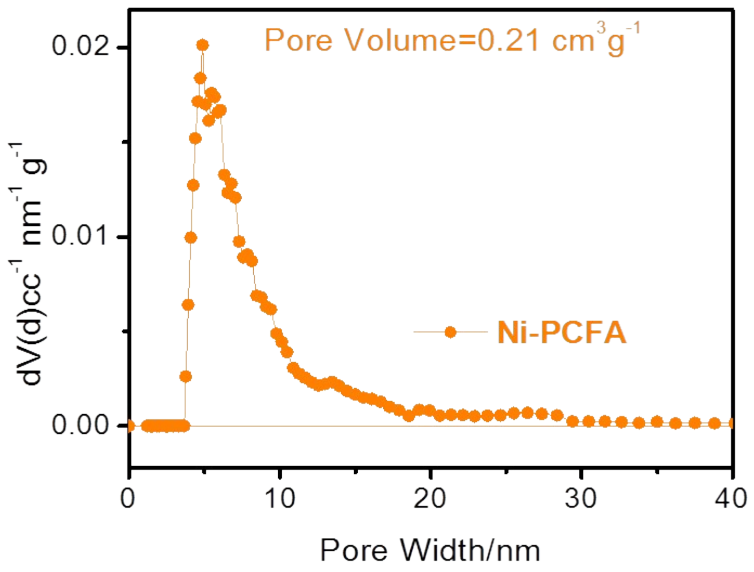

Figure S6. Pore-width distribution of Ni-PCFC.

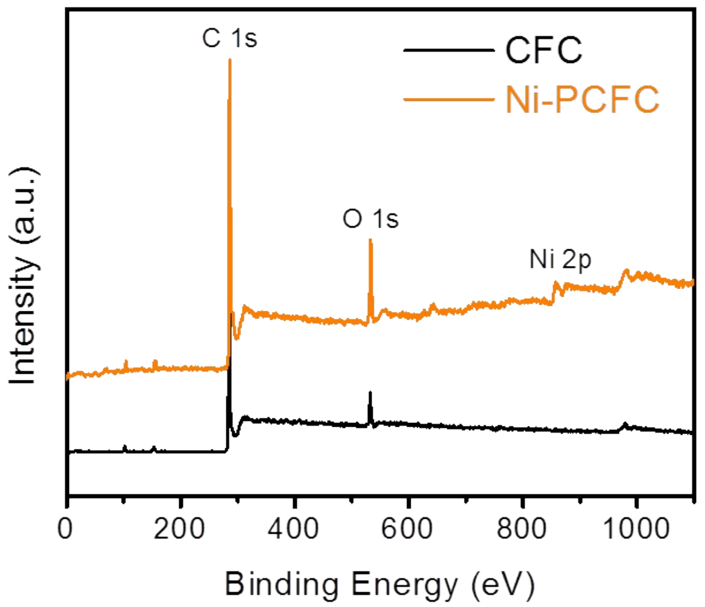

Figure S7. XPS survey spectra of the CFC and Ni-PCFC.

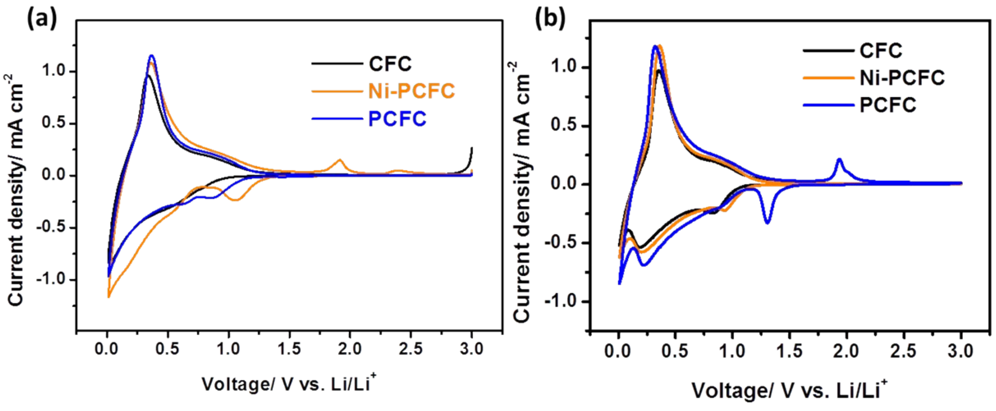

Figure S8. 1st and 2nd CV profile of CFC, Ni-PCFC and PCFC electrodes.

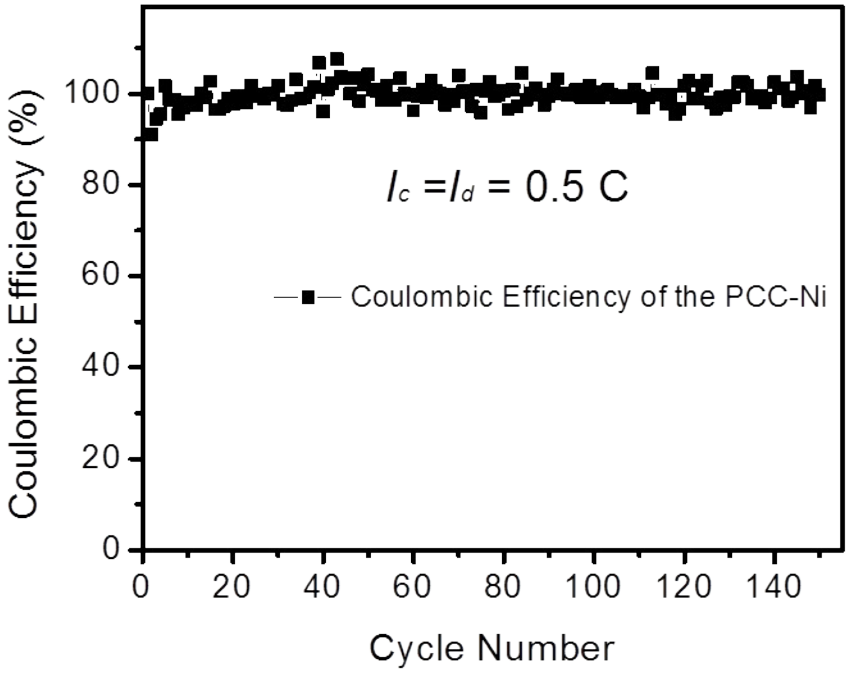

Figure S9. Coulombic efficiency of the cyclic stability of Ni-PCFC electrode at 0.5C from Figure 4c.

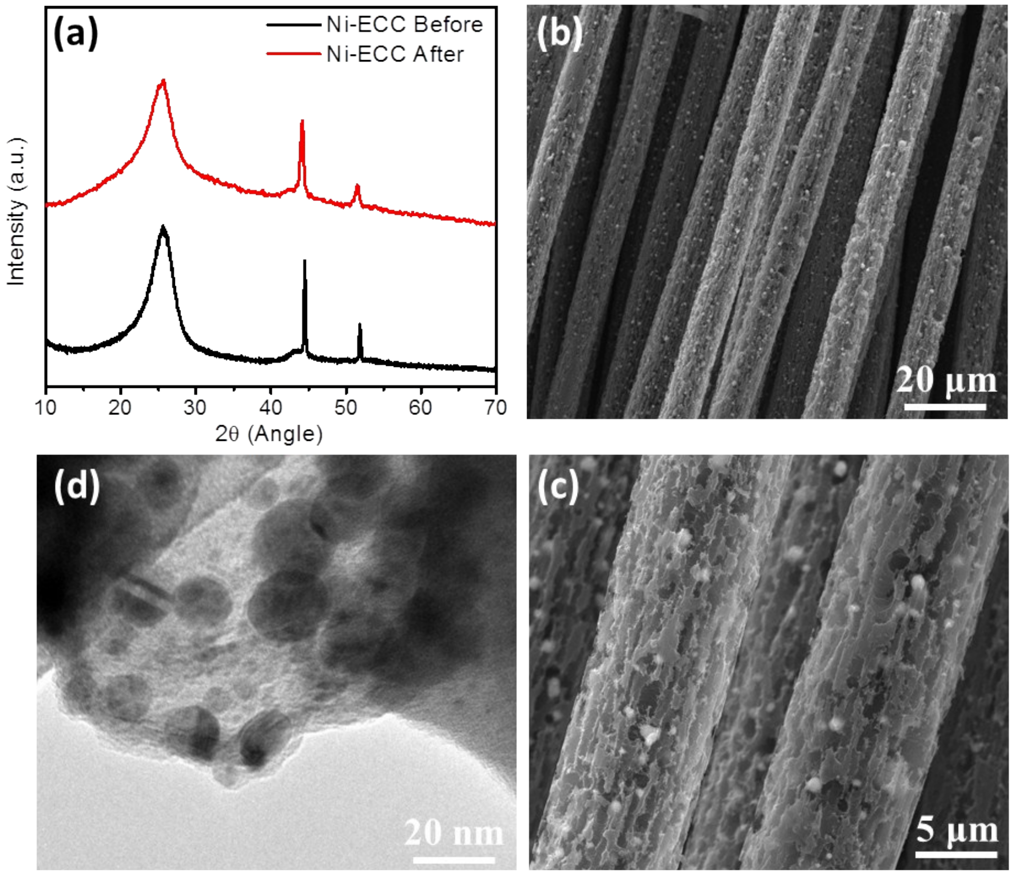

Figure S10. (a) XRD pattern of Ni-PCFC before and after electrochemical reaction. (b) Low magnification and (c) magnified SEM images of Ni-PCFC after electrochemical reaction. (d) HRTEM image of Ni-PCFC after electrochemical reaction.
